# Aβ immunotherapy drives immune activation and cerebrovascular damage in a mouse model of Alzheimer’s Disease

**DOI:** 10.1002/alz.085423

**Published:** 2025-01-03

**Authors:** Xavier Taylor, Harun Noristani, Griffin Fitzgerald, Herold Oluoch, Nick Babb, Tyler McGathey, Justin T. Hole, Pascale Lacor, Ronald B. DeMattos, Yaming Wang, Neuroscience Discovery, Lilly Research Laboratories, Eli Lilly and Company, Indianapolis, IN, 46285, USA

**Affiliations:** ^1^ Eli Lilly and Company, Indianapolis, IN USA; ^2^ Imaging Research and Development, Lilly Research Laboratories, Indianapolis, IN USA

## Abstract

**Background:**

Anti‐amyloid‐β (Aβ) immunotherapy trials have shown amyloid‐related imaging abnormalities (ARIA) as the most common and serious adverse events linked to pathological changes in cerebral vasculature. Nevertheless, the mechanisms underlying how amyloid immunotherapy triggers vascular damage, increases vascular permeability, and results in microhemorrhages remains unclear. Notably, activation of perivascular macrophages and infiltration of peripheral immune cells have been implicated in regulating cerebrovascular damage. However, further research is needed to understand the immediate downstream consequences of macrophage activation that exacerbate CAA‐related vascular permeability and microhemorrhages associated with amyloid immunotherapy.

**Methods:**

This study investigates immune responses induced by amyloid‐targeting antibodies and CAA‐induced microhemorrhages using RNA in situ hybridization, histology and digital spatial profiling in an Alzheimer’s disease (AD) mouse model of microhemorrhage.

**Results:**

In the present study, we demonstrate anti‐Aβ (3D6) immunotherapy induces robust vascular inflammation and profound smooth muscle cell loss, leading to vascular damage and loss of blood‐brain barrier (BBB) integrity. Additionally, digital spatial profiling reveals robust recruitment of peripheral immune cells encompassing T and B‐ lymphocytes encircling vascular amyloid deposits. Finally, RNA in‐situ hybridization coupled with immunohistochemistry identifies 2 distinct subsets of macrophages encompassing tissue‐resident and monocyte‐derived macrophages around vascular amyloid deposits after Aβ immunotherapy demonstrating the multifaceted roles of immune activation and vascular damage in response to Aβ immunotherapy.

**Conclusions:**

In summary, our study establishes a significant link between CAA‐Aβ antibody immune complex formation, macrophage activation, vascular damage leading to impairment of BBB integrity. Nevertheless, the full implications of this phenomenon on the susceptibility to microhemorrhages needs to be explored further. Therefore, additional investigations are needed to determine the potential preferential roles played by tissue‐resident and monocyte‐derived macrophages surrounding vascular amyloid deposits, unraveling the intricate interplay between immune activation, vascular damage and accelerated smooth muscle cell loss in response to Aβ immunotherapy.

